# Sexual activity after short-stem total hip arthroplasty. Does stem size matter?

**DOI:** 10.1007/s00402-022-04614-y

**Published:** 2022-09-17

**Authors:** Georg Hauer, Patrick Sadoghi, Maria Smolle, Sabrina Zaussinger, Joerg Friesenbichler, Andreas Leithner, Werner Maurer-Ertl

**Affiliations:** Department of Orthopaedics and Trauma, Medical University of Graz, Auenbruggerplatz 5, 8036 Graz, Austria

**Keywords:** Total hip arthroplasty, Total hip replacement, Short-stem, Return to sexual activity

## Abstract

**Background:**

Sexual activity is an important component of quality of life. To date, no studies have examined the impact of stem design on return to sexual activity (RTS) and quality of sex life after total hip arthroplasty (THA).

**Methods:**

A questionnaire was designed to assess preoperative and postoperative sexual habits, joint awareness and physical activity in working-age patients undergoing short-stem (*n* = 176) or straight-stem (*n* = 97) THA.

**Results:**

No differences were noted in time until RTS based on the stem design (short stems vs. straight stems; 6 [IQR: 4–10] vs. 6 [4–10] weeks; *p* = 0.996). Multivariate analysis revealed that higher patient BMI (*p* = 0.04), female gender (*p* < 0.001) and lower FJS-12 (*p* = 0.006) were significantly associated with delayed RTS. Improved hip mobility and reduced pain mainly contribute to improved quality of sexual activity postoperatively.

**Conclusion:**

This study, hence, indicates that stem design has no impact on time until resumption of sexual activity in patients < 65 years. Female obese individuals who are aware of their artificial joint in daily life are at increased risk of delayed RTS after surgery.

**Level of evidence:**

Level III, retrospective cohort study.

## Introduction

Today's total hip arthroplasty (THA) receivers are demographically different than in the past [5]. Initially designed for elderly patients with the objective to relieve pain, the working-age population now account for an increasing percentage of the patients receiving hip replacement surgery [2, 15, 20]. This trend looks set to continue as demand for THA is predicted to grow 174% by 2030 [15]. Along with the demographic change, the demands on the operation have also changed. Active patients expect not only to improve pain and mobility, but also to be able to do sports, return to work and engage in sexual activity. Sexual life is a vital dimension in overall quality of life and successful return to sexual activity (RTS) has been specifically identified as a high priority in patients undergoing THA [6, 12, 14, 18, 19]. If such increased expectations are not met, there may be dissatisfaction with the perceived outcome [24].

In recent years, short-stems in THA have gained increasing popularity [2]. In young, active patients, short-stems are increasingly regarded as implants of first choice allowing mini-invasive exposure and bone sparing approaches [8, 16]. Compared to conventional straight-stem, new designs also aim at the preservation of proximal bone stock, by reducing stress-shielding due to periprosthetic bone remodeling, should revision surgery become necessary [9, 21, 25]. Clinical short- to mid-term results are encouraging, however, there is a lack of literature regarding return to sexual function and its frequency and quality following short-stem THA. Moreover, it is uncommon for surgeons and patients to discuss this topic during consultation [13, 22, 26].

This study, therefore aimed, to explore the association between resumption and quality of sexual activity and stem design in the working-age population. The second purpose was to evaluate risk factors that are associated with delayed RTS.

## Methods

After local ethics committee approval was obtained for this study, we reviewed the medical records of all patients undergoing elective THA between November 2013 and August 2019. Patients were eligible for the study if they (1) were below the retirement age (male < 65, female < 62) at the time of their THA, (2) were at least six months after surgery, and (3) had diagnosis of noninflammatory arthritis (osteoarthritis, posttraumatic arthritis, avascular necrosis, or hip dysplasia). The age maximum of 62–65 years was instituted to focus the study on the working-age population. Exclusion criteria were individuals who were less than 6 months after surgery, had one-stage bilateral THA or hip replacement due to femoral neck fracture or tumour or had received a previous THA or total knee arthroplasty (TKA) within the last 6 months. Patients who were below the retirement age, but already retired at the time of THA, were also excluded from analysis.

There were 581 patients who fit the eligibility criteria for this study, including 337 individuals after short-stem THA and 244 after straight-stem THA. Digital preoperative planning, surgeon’s preference and bone quality were decisive for the choice between short-stem and straight-stem. In all short-stem patients, the cementless, metaphyseal-anchoring, femoral-neck preserving ANA NOVA Alpha Shaft Proxy *(ImplanTec)* was used. All patients in the straight-stem group received a cementless Corail stem *(Johnson&Johnson).* Both stems were combined with cementless press-fit cups (ANA NOVA Alpha Cup*, Implantec* or Pinnacle Cup, *Johnson&Johnson*) with a ceramic–ceramic bearing couple. All THAs were carried out or supervised by experienced surgeons at our hospital using the minimally invasive, modified anterolateral approach. The rehabilitation protocol included full weight bearing using two crutches immediately postoperatively. No specific recommendations regarding return to sexual activity were made.

A self-designed questionnaire was posted to those patients who fulfilled the inclusion criteria. A reminder call was made to those patients who did not return the questionnaires within six weeks. If there was no response for another six weeks, they were excluded. Questionnaires asked patients to describe their current sexual function, as well as recall details regarding preoperative sexual function and habits. The questionnaire also compared preoperative and postoperative quality and quantity of sexual activity and included a question about the time until RTS. It also contained items related to employment status, pain before and after surgery, activity level and joint awareness. The patients’ activity level was measured with the University of California, Los Angeles (UCLA) activity scale [1]. The Forgotten Joint Score-12 (FJS-12) was asked to assess patients’ awareness of their artificial joints in everyday life [3]. The FJS-12 ranges from 0 to 100, the higher the score, the more natural or “forgotten” the joint. Demographic data such as age, gender, and clinical data such as body-mass index (BMI), type of stem and previous joint replacements were collected from the patients’ medical records.

**Fig. 1:**
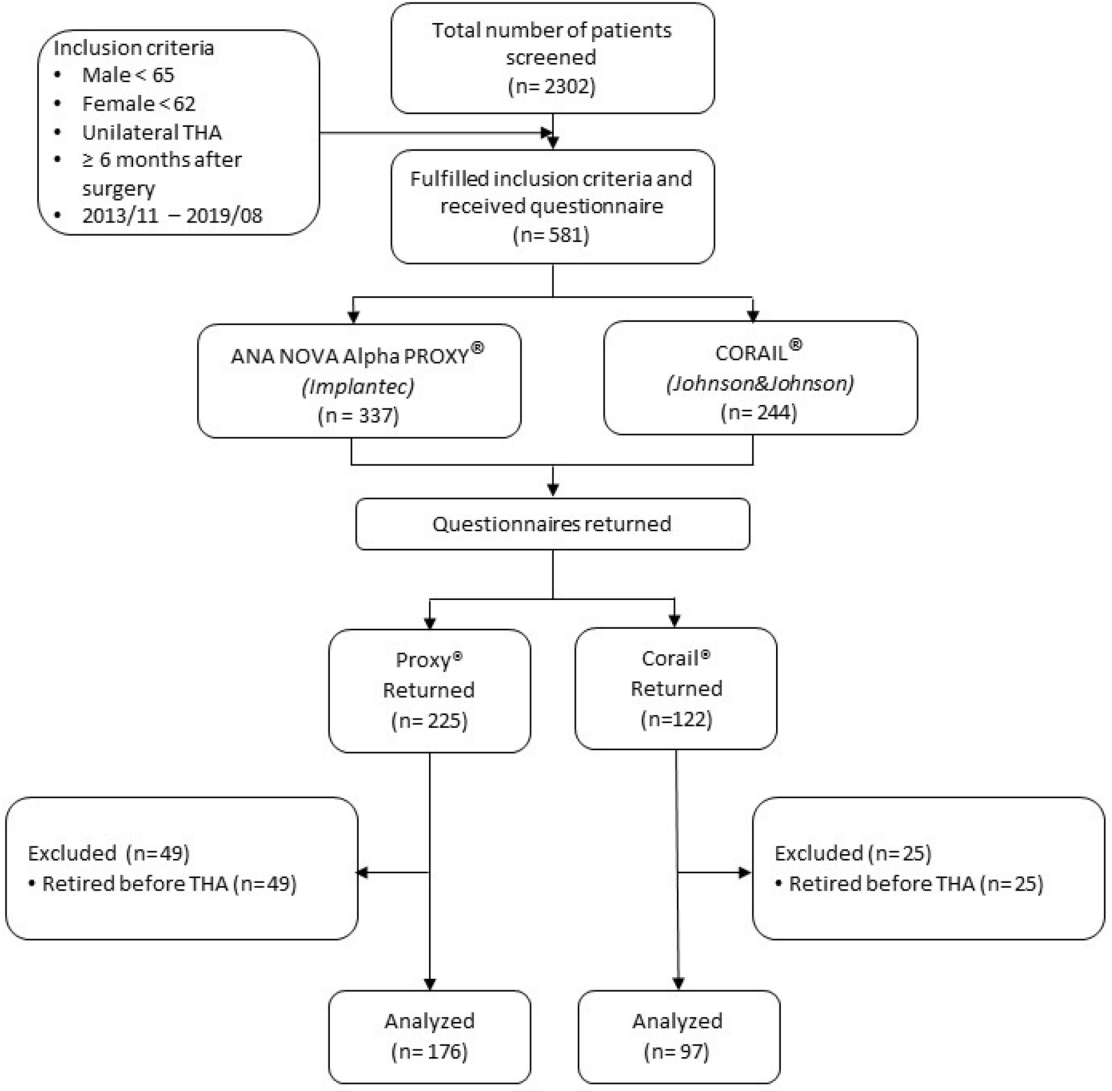
Patient flow chart on study inclusion

We received responses from 347 patients and no response from 234 patients. Of the 347 patients who responded, 74 were excluded since they were already retired at the time of surgery. Figure 1 shows the distribution of the patients who were included and excluded and their characteristics.

### Statistical analysis

Statistical analyses were performed with Stata Version 13.0 (StataCorp, College Station, US). Differences in categorical variables between groups were assessed with Chi-squared tests. Wilcoxon-rank-sum tests and (paired) *t* tests were performed to analyse differences between groups for non-parametric and parametric variables, respectively.

Multivariate linear regression analysis was performed to estimate the influence of parameters on RTS. For this approach, a stepwise backward selection model, excluding all variables with a *p* value of < 0.05, was constructed. For all analyses, a *p* value of < 0.05 was considered statistically significant.

## Results

### Descriptive analysis

Of the 273 patients included, 176 had received a short-stem (Proxy®; 64.5%) and 97 a straight-stem THA (Corail®; 35.5%). Demographic baseline characteristics between the groups, preoperative sexual habits and type of THA are illustrated in Table 1. Mean time from THA to completion of the questionnaire was 30.9 months (± 20.2 months), with a significantly shorter interval for patients with a short-stem THA in comparison to a straight-stem THA (20.5 ± 10.7 months vs. 50.0 ± 19.4 months; *p* < 0.001). Whilst median preoperative UCLA activity score was comparable between short- and straight stem-group (4 [IQR: 3–7] vs. 5 [3–7]; *p* = 0.708), median postoperative UCLA activity score was significantly better in patients with short-stem THA (7 [6–9] vs. 7 [5–8]; *p* = 0.018). Median postoperative FJS-12 was 72.9 [IQR: 47.9–89.6] with no significant difference between short- and straight-stem groups (75 [IQR: 52.1–89.6] vs. 66.7 [IQR: 45.8–89.6]; *p* = 0.197).

**Table 1:** Differences in demographic variables between groups

		Male	Female	Missing (*n*)	*p *value
Age at surgery (mean, in years)		55.5 ± 6.0	52.7 ± 5.9	2	< 0.001
BMI (mean)		28.4 ± 5.2	27.5 ± 6.0	2	0.221
Preoperative sexual habit	Inactive	13 (9.6%)	21 (18.9%)	27	0.036
	Active	122 (90.4%)	90 (81.1%)		
Type of THA	Short stem	99 (68.3%)	77 (60.2%)	0	0.162
	Straight stem	46 (31.7%)	51 (39.8%)		

### Sexual habits

**Fig. 2:**
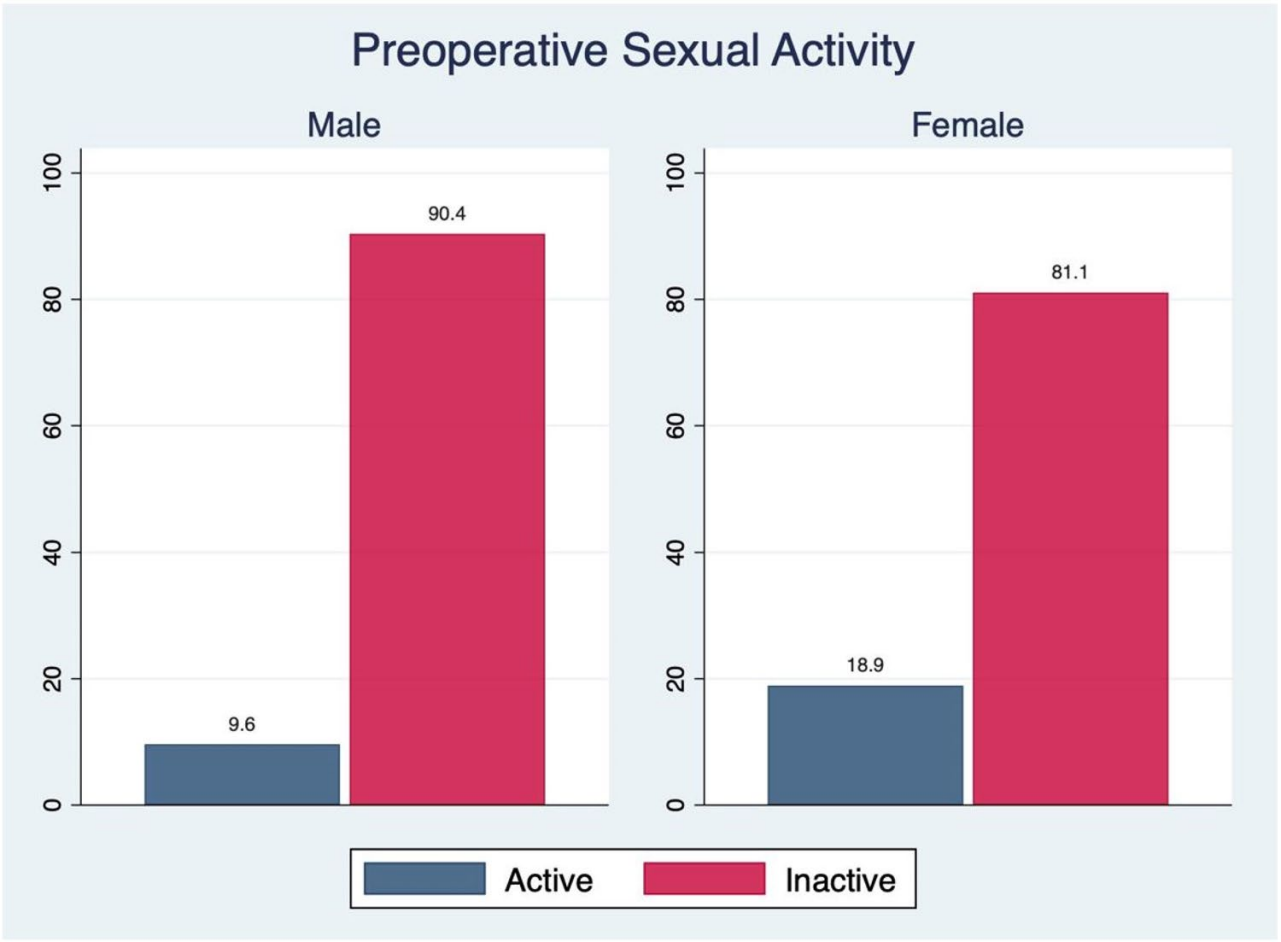
Preoperative sexual activity between males and females

Preoperatively, 9.6% of male (*n* = 13) and 18.9% (*n* = 21) of female patients had been sexually inactive (*p* = 0.036; Table 1; Fig. 2). Of those patients being sexually active prior to surgery, 61.6% (*n* = 127) reported having pain associated with their diseased hip limiting their sexual habits. Again, female patients more often reported hip-associated pain (*n* = 65; 73.0%) limiting sexual activity in comparison to male patients (*n* = 62; 52.1%; *p* = 0.002). Of note, the duration of hip-associated pain interfering with sexual activity was comparable between males (32 weeks [IQR: 18–60 weeks]) and females (45 weeks [IQR: 13–52 weeks]; *p* = 0.670).

**Fig. 3:**
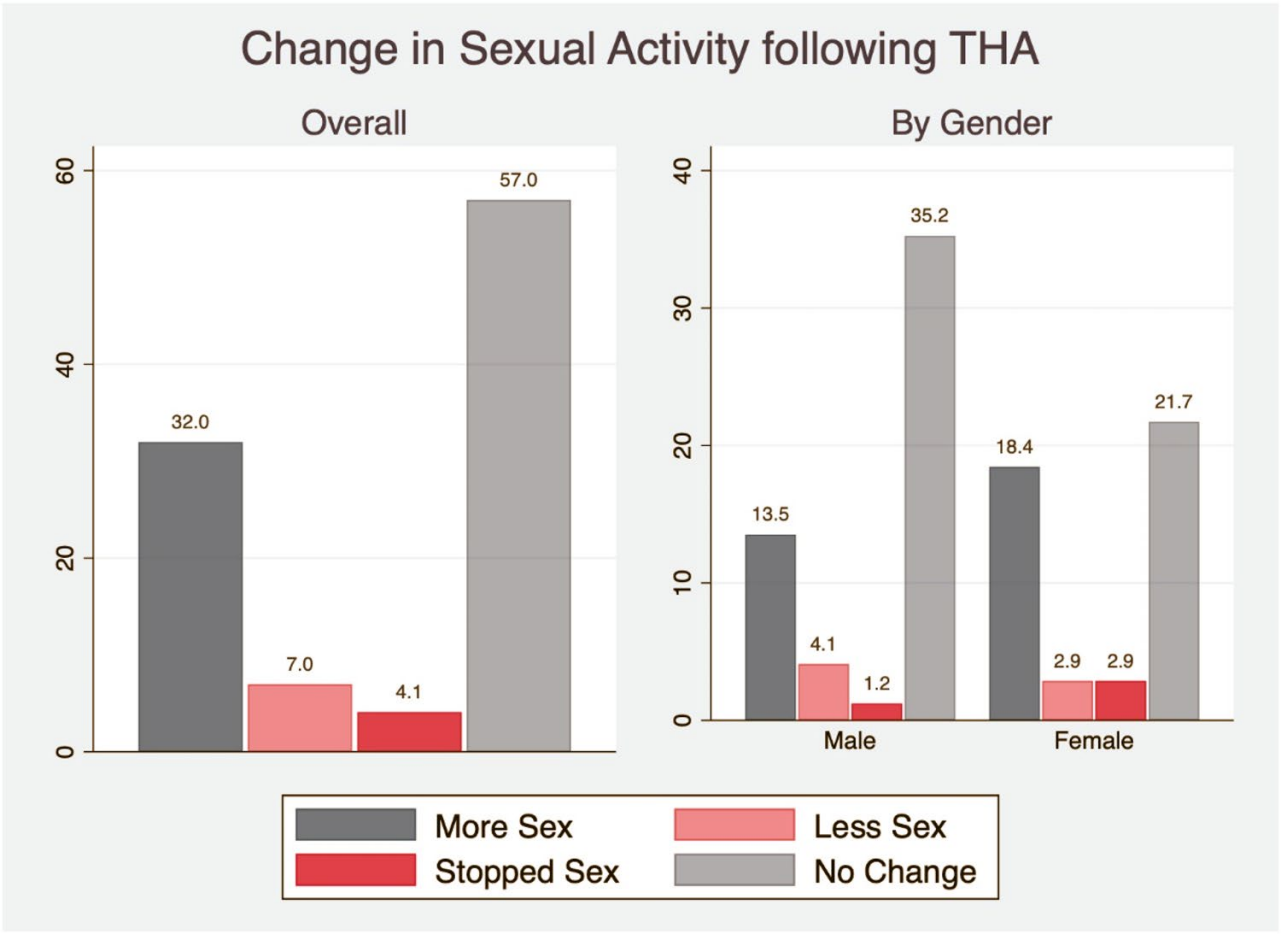
Change in sexual activity following THA by gender

Figure 3 illustrates the change in sexual activity following THA. The change was significantly different between males and females, with 40.2% of female patients (*n* = 45) reporting having more sexual intercourse postoperatively, in comparison to 25.0% (*n* = 33) of male patients only (*p* = 0.017; Fig. 3). The median time until restarting sexual activity was 6 weeks [IQR: 4–10 weeks], with male patients resuming sexual activity significantly earlier in comparison to females (5 weeks [IQR: 3–8 weeks]) vs. 8 weeks [IQR: 5–12 weeks]; *p* < 0.001). No significant difference between the two groups were observed [short stems vs. straight stems; 6 (IQR: 4–10] vs. 6 [4–10] weeks; *p* = 0.996].

### Quality of sexual activity

Improvement in hip mobility (*n* = 103) was the most frequent reason given by patients to improve quality of sexual activity, followed by reduced hip pain (*n* = 89) and less fear about the diseased hip (*n* = 45). These reasons were independent from gender (male vs. female; *p* = 0.489, *p* = 0.557, *p* = 0.583, respectively) and stem design (short-stem vs. straight-stem; *p* = 0.771, *p* = 0.853, *p* = 0.724, respectively).

### Factors associated with delayed return to sexual activity

**Fig. 4:**
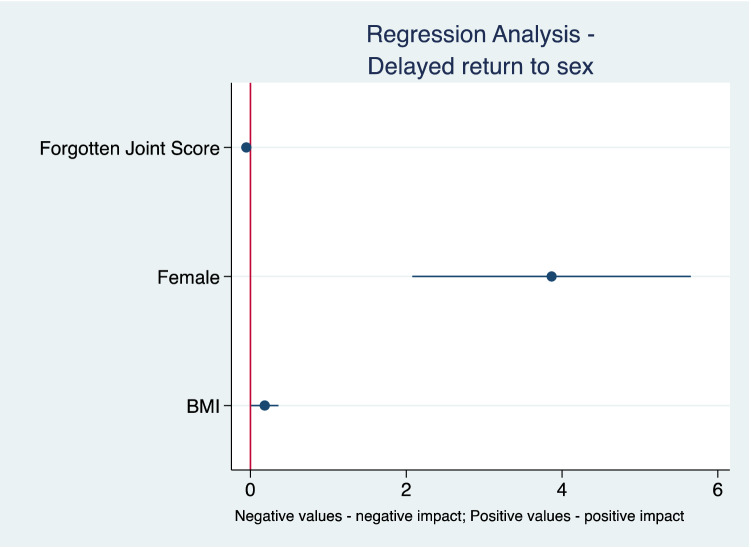
Graphical presentation of multivariate linear regression analysis

In the multivariate linear regression analysis, after excluding pre- (*p* = 0.667) and postoperative UCLA (*p* = 0.240), pre- (*p* = 0.212) and postoperative VAS (*p* = 0.902), short- vs. straight-stem group (*p* = 0.879), quality of sexual activity (*p* = 0.805), preoperative diagnosis (*p* = 0.718), interval from THA to questionnaire (*p* = 0.231), and age at surgery (*p* = 0.397) with backward stepwise selection, patient BMI (*p* = 0.040), gender (*p* < 0.001) and FJS-12 (*p* = 0.006) were significantly associated with delayed RTS (Table 2; Fig. 4).

**Table 2:** Multivariate linear regression analysis assessing variables influencing time period to restarting sex

Return to sexual activity		Coefficient	SE	95% CI [Lower; Upper]	*p* value
Forgotten-joint-score-12		-0.05	0.02	[− 0.09; − 0.02]	0.006
BMI		0.18	0.09	[0.00; 0.36]	0.040
Gender	Male	Ref			< 0.001
	Female	3.87	0.91	[2.08; 5.65]	
Constant		4.47	3.03	[− 1.50; 10.44]	0.141

Age at surgery (*p* = 0.405); short- vs. straight-stem group (*p* = 0.879); pre- (*p* = 0.667) and postoperative UCLA (*p* = 0.240); pre- (*p* = 0.212) and postoperative VAS (*p* = 0.902); preoperative diagnosis (*p* = 0.718); quality of sex (*p* = 0.805); interval THA-questionnaire (*p* = 0.231)

*SE* standard error, *CI* confidence interval

## Discussion

Successful THA has been reported to improve sexual satisfaction and performance in most patients [6, 11, 14, 17–19]. RTS is an important goal following joint arthroplasty surgery and an essential component of quality of life [19, 23]. In the current literature, very little is known about the role of THA components on postoperative sexual activity, especially with emphasis on the femoral stem. Nunley et al. [19] described no statistically significant differences in any sexual function outcome categories based on bearing surface, femoral head size and comparison between standard THA and surface replacement surgery. The current study is novel in which it assessed the influence of stem design on postoperative sexual activity. The most important finding was that most patients below the retirement age returned to their baseline or higher level of sexual activity. The median time of resuming sexual intercourse was about six weeks and short-stems were not associated with earlier RTS than conventional straight-stems. A better hip mobility and reduced pain are key factors for improved quality of sexual activity postoperatively.

The time until resumption of sexual activity was similar to those previously reported and is within the recommended period of 1–3 months based on the consensus opinion from experienced joint replacement surgeons [6, 14]. In general, one month is the minimum time required for the soft tissue to heal and prevent early dislocation [7, 18].

Our study found that more than half of the patients before THA experienced diminished sexual quantity and satisfaction because of hip pain. Again, notable differences between men and women were observed. Female patients were more likely to complain of pain as limiting amount and quality of sexual intercourses than men. Gender-related differences could be explained by the fact that sexual positions for women require extreme hip range of motion (ROM), while sexual positions for men require less mobility [4].

In our study, the vast majority of hip arthroplasty patients (96%) < 65 years were sexually active postoperatively. Compared with patients’ state preoperatively, results from our study indicate that 32% of patients may have more sexual intercourse after THA, whereas 57% may expect no change in their sexual activity because of surgery, 7% may have less sexual activity, and 4% may stop having sexual activity. Once again, we found discrepant results between men and women before and after surgery. Significantly more women than men reported having more sexual activity postoperatively. This was not a surprising finding because women reported significantly more complaints of pain before surgery and a significant reduction of quality compared to men after hip replacement. The enhanced quality was mainly attributed to greater mobility and less pain. In addition, Fu et al. [10] observed similar findings, who documented that changes in the hip ROM have been significantly correlated with improvements in sexual activity.

There is little in the literature regarding which factors influence the time of RTS. The results of multivariate regression analysis in the current study demonstrate that BMI, gender and joint awareness have a significant effect on RTS. A lower FJS-12, female sex and a higher BMI have impaired the time of resuming sexual activity. The FJS-12 measures the ability for a patient to forget about their artificial joints in everyday life. The ultimate goal after THA is for a patient to be “unaware” of the prosthetic joint [3]. Our findings suggest, that the sooner patients “forget” about their new hip prosthesis, the earlier they start to resume sexual activity.

There are certain limitations to the present study. First of all, the incomplete response rate, which could be explained by the sensitive nature of the questions asked. Second, factors like relationship status, general or mental health and other environmental factors that affect sexual activity and frequency were not evaluated. Third, we contacted some patients up to six years postoperatively and their recollection of events and in particular their time until RTS, may not be entirely reliable.

## Conclusion

In summary, the majority of working age patients return to sexual activity after THA within a short-time period and stem design has no impact on time until resumption of sexual activity. Female obese individuals who are aware of their artificial joint in daily life are at increased risk of delayed RTS after surgery. We believe that this study provides valuable data to be used by orthopaedic surgeons when discussing RTS after short-stem THA with their patients.
